# Antibody or Anybody? Considering the Role of MRGPRX2 in Acute Drug-Induced Anaphylaxis and as a Therapeutic Target

**DOI:** 10.3389/fimmu.2021.688930

**Published:** 2021-11-19

**Authors:** Graham A. Mackay, Nithya A. Fernandopulle, Jie Ding, Jeremy McComish, Paul F. Soeding

**Affiliations:** ^1^ Department of Biochemistry and Pharmacology, The University of Melbourne, Parkville, VIC, Australia; ^2^ Department of Clinical Immunology and Allergy, The Royal Melbourne Hospital, Parkville, VIC, Australia; ^3^ Department of Anaesthesia and Pain Medicine, The Royal Melbourne Hospital, Parkville, VIC, Australia

**Keywords:** anaphylaxis, mast cells, drug hypersensitivity, MRGPRX2, IgE (immunoglobulin E)

## Abstract

Acute anaphylaxis to small molecule drugs is largely considered to be antibody-mediated with immunogloblin E (IgE) and mast cell activation being key. More recently, a role for drug-reactive immunoglobulin G (IgG) with neutrophil activation has also been suggested, at least in reactions to neuromuscular blocking agents (NMBAs). However, the mast cell receptor MRGPRX2 has also been highlighted as a possible triggering mechanism in acute anaphylaxis to many clinically used drugs. Significantly, MRGPRX2 activation is not dependent upon the presence of drug-recognising antibody. Given the reasonable assumption that MRGPRX2 is expressed in all individuals, the corollary of this is that in theory, anybody could respond detrimentally to triggering drugs (recently suggested to be around 20% of a drug-like compound library). But this clearly is not the case, as the incidence of acute drug-induced anaphylaxis is very low. In this mini-review we consider antibody-dependent and -independent mechanisms of mast cell activation by small molecule drugs with a focus on the MRGPRX2 pathway. Moreover, as a juxtaposition to these adverse drug actions, we consider how increased understanding of the role of MRGPRX2 in anaphylaxis is important for future drug development and can complement exploration of this receptor as a drug target in broader clinical settings.

## Introduction, Overview and Significance

The risk of adverse drug reactions such as anaphylaxis, whilst rare, remains a serious concern. In susceptible individuals, specific drug exposure may trigger a sudden life-threatening reaction, and unless a history of previous hypersensitivity exists, this response is mostly unpredictable. Even within the perioperative setting, where facilities for resuscitation are optimal, drug-induced anaphylaxis still causes a significant incidence of patient injury and mortality (1–3). Here we examine recent mechanistic advances in the understanding of drug-induced anaphylaxis in humans, with a focus on the critical role played by mast cell activation and the role of the Mas-related G protein-coupled receptor X2 (MRGPRX2). It is noteworthy that there have been several recent excellent and comprehensive reviews of drug hypersensitivity and MRGPRX2 involvement in human disease that complement the present article (4–7).

Commonly, mechanisms of drug-induced acute anaphylaxis are classified as either being ‘antibody (IgE)-dependent’ or ‘other’ depending upon the clinical diagnostic workup. With the identification of MRGPRX2, the activation of this receptor has emerged as a viable explanation to classify these previously mechanistically uncertain cases (reported to be around 30% of events). Studies in mice clearly support the involvement of MrgprB2 (the murine homologue of MRGPRX2) in drug-induced anaphylaxis to polybasic compounds such as NMBAs (8). However, unsurprisingly, this is more challenging to prove in humans. Whilst skin injection site reactions are observed very commonly to certain known MRGPRX2 activators (e.g. icatibant), consistent with the high expression of MRGPRX2 in mast cells in this location (discussed later), systemic anaphylactic responses to such compounds have not been reported (9, 10). To date, there is no means of unambiguously attributing a clinical event of drug-induced acute anaphylaxis to MRGPRX2 activation.

From a patient perspective, defining the role of MRGPRX2 is important as if confirmed, it may provide predictive, preventative and therapeutic strategies for drug-induced anaphylaxis. Moreover, the pharmaceutical industry is increasingly examining drug agonism at MRGPRX2 in their pre-clinical drug candidate evaluations (11, 12). In one such study, around 20% of a drug-screening chemical library was shown to be MRGPRX2-activating (11). Presumably, such pre-clinical screening could be used to discard, or at least de-prioritise, drug candidates/leads. Whilst this might be seen as improving drug safety, using icatibant as an example, it may result in future life-saving therapeutics being discarded unnecessarily. As such, defining the true clinical role of MRGPRX2 in drug-induced anaphylaxis has wide-sweeping importance.

### Lost in Translation: Discriminating Antibody-Dependent and MRGPRX2-Dependent Drug-Induced Anaphylaxis

Conclusive evidence that MRGPRX2 activation is a primary mechanism in drug-induced anaphylaxis continues to be a clinical challenge. Here we largely compare IgE-dependent with MRGPRX2-dependent reactions although we acknowledge that this is oversimplistic and IgE and mast cell-centric. For instance, IgG-dependent reactions involving neutrophils have been reported, initially in mice, but more recently suggested to be important to drug-induced anaphylaxis in humans, at least with NMBAs (4, 13, 14). Involvement of the mast cell activating complement anaphylatoxins (C3a/C5a) in immune-mediated anaphylaxis has also been reported (13, 15).

Whilst it is well established that IgE is responsible for the majority of drug-induced acute anaphylaxis, new information on this pathway is also arising. A recent study has shown a role for a subset of T follicular helper cells in the production of high-affinity IgE to allergens (16). Whilst it is unclear if this extends to small molecule drugs acting as haptens, it nonetheless suggests that ‘quality over quantity’ might be important for IgE-dependent anaphylaxis, which has implications for the identification of culprit pathways. For instance, it is possible that the inability to attribute drug anaphylaxis to IgE is a result of an inability to detect it rather than the lack of its presence. Drug-‘specific’ serum IgE testing is however commonly incorporated into diagnostic algorithms. For this, prototypic drugs that display chemical structural features of common culprit agents (e.g. morphine, penicilloyl conjugates) are often used in testing. This approach not only lacks sensitivity (17) but also specificity as it might ignore IgE that recognises diverse drug epitopes. Whilst some clinical centres use a wider range of potential culprit drugs in screening, this is relatively uncommon and thus assays to detect true drug-specific IgE (or indeed IgG) are needed.

Whilst it was originally thought that changes to serum levels of tryptase could be used to discriminate between IgE and MRGPRX2-dependent reactions, recent work has suggested against this (18, 19) which supports *in vitro* studies that report non-IgE-dependent secretion of tryptase (20, 21).

The use of the skin prick and intra-dermal tests are common in clinical investigations to identify culprit anaphylaxis-inducing drugs. Indeed, morphine/codeine have such predictable general reactivity in intradermal testing that they are often used as a positive control stimuli. This approach is thus clearly not a discriminatory tool between IgE- and MRGPRX2-dependent pathways as both could be active in skin mast cells.

More recently, addition of the basophil activation test (BAT) has been suggested as a discriminatory assay in mechanistic attribution of drug-induced anaphylaxis (22). The discriminatory utility of this *ex vivo* assay is based on the observation that basophils, in general, are not thought to have functional expression of MRGPRX2. Basophil activation by NMBAs therefore would strongly suggest an IgE-dependent mechanism. However, a recent study has suggested functional expression of MRGPRX2 on basophils (23), although this has been suggested to relate to basal activation of the cells and consequent expression of a normally intracellular pool of receptor (24). A broader discussion of the potential utility of the BAT approach in identifying non-IgE-dependent pathways in drug-induced anaphylaxis has been recently published (22).

The possibility of using the known differences in the FcεRI and MRGPRX2 signaling pathways (25) also has potential to resolve the IgE *vs* MRGPRX2 conundrum. Bruton’s tyrosine kinase (Btk) inhibitors, clinically used to treat leukemia, have been shown to be powerful inhibitors of IgE-dependent human mast cell activation (26). Importantly, based on the receptor’s signalling cascade, these approved drugs would not be predicted to affect the MRGPRX2 pathway. In theory, Btk inhibitors could be used locally during skin challenge testing, and thereby provide mechanistic evidence for the pathway underpinning anaphylaxis. The feasibility and safety of this approach has already been partially established using the Btk-inhibitor ibrutinib (27). Other approved compounds such as fostamatinib, a spleen tyrosine kinase (Syk) inhibitor, could be used in a similar way. Whilst speculative, such extension of skin-prick testing is clinically feasible although, from an ethical and safety perspective, would be easier to incorporate into existing *ex vivo* approaches such as in BAT analysis and/or in studies using skin biopsies.

**Figure 1:**
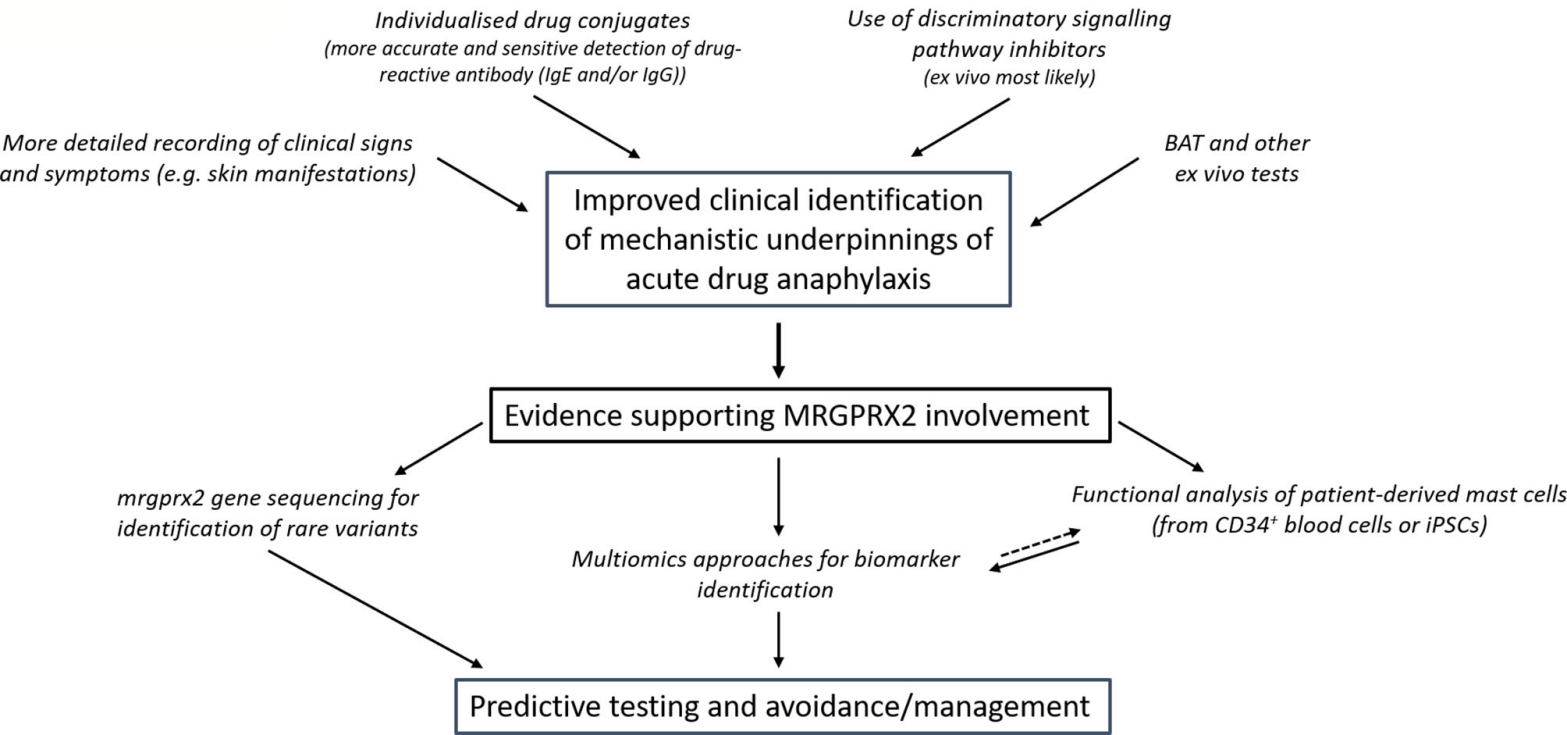
Proposed approaches to overcome the current deficiencies in clinical discrimination of patients who suffer MRGPRX2-dependent anaphylaxis and their prospective value. Better defining patients who likely suffered MRGPRX2 dependent anaphylaxis enables more focused, powerful and feasible research that can be used prospectively in predictive testing. The acute and commonly severe nature of drug-induced anaphylaxis means that discrimination between the pathways would likely have little consequence to the present-day management of patient symptoms. However, further comparative insights might highlight approaches that could perhaps provide more discrete benefit. (BAT- basophil activation test).

Accurate clinical differentiation of a likely MRGPRX2-dependent subgroup of patients at risk of severe reactions to a given medication would enhance prospects of developing predictive biomarkers (**Figure 1**). What such a biomarker might be remains elusive, but we consider some of the possibilities and gaps in understanding below.

### Elevated and/or Expanded Expression or Function of MRGPRX2 in Mast Cells

Mast cells mature into their characteristic highly granular form within tissues. However, variation in the type and levels of soluble factors and extracellular matrix proteins results in differential mast cell gene expression patterns and consequent functional heterogeneity to drug stimulation (28–30). More recently, antibody tools and transcriptomic and proteomic approaches have characterised this heterogeneity more comprehensively at the molecular level and provided alternative approaches for quantifying mast cells and MRGPRX2 expression in tissues (31–33). However, responses to compounds/agents now known to be direct activators of MRGPRX2 (e.g. compound 48/80) can also be used as a surrogate marker of the functional expression of MRGPRX2. Using these combined approaches, MRGPRX2 expression is particularly pronounced and consistently found in primary human mast cells isolated from the skin and fat with more variable expression in the gut and lung (32) that reflects the well-reported heterogeneity of mast cells in the latter organs (29). There is also evidence of MRGPRX2 functional expression in the heart and synovial tissue (20, 34). Thus, while skin mast cells are undoubtedly a focal point, as observed with the common injection reactions seen to some MRGPRX2 activating drugs, mast cells in other locations also have the potential to be triggered by the same compounds and may therefore contribute to systemic adverse responses to drugs.

Given the strong expression of MRGPRX2 in skin mast cells, it might be expected that cutaneous symptoms would be overt in putative MRGPRX2-dependent anaphylaxis. However, this has to our knowledge not been formally reported and might be complicated by core hypotension and the rapid administration of a variety of life-sustaining drugs upon signs of anaphylaxis.

It is possible that an elevated or more diverse tissue expression of MRGPRX2, perhaps associated with disease, may enhance an individual’s susceptibility to drug-induced anaphylaxis. To our knowledge there are no published studies that examine mast cell MRGPRX2 expression in the context of acute drug-induced anaphylaxis. These studies are challenging as given the highly selective expression of MRGPRX2 to mature, tissue-resident mast cells, blood cell transcriptomics approaches will likely not be optimal. Skin biopsies would be much more useful in this regard especially with the increasing use of single cell genomic approaches. Furthermore, whilst transcriptomic approaches would seem the best approach to resolve this, studies have shown that MRGPRX2 mRNA levels are not a good measure of surface expression of the receptor (24, 35). This suggests non-transcriptional factors may also dynamically regulate MRGPRX2 surface levels, although the regulators of this process are unclear.

Regulators of MRGPRX2 expression, at the transcriptional and/or post-transcription levels remain unclear. Chronic IL-6 treatment during the generation of blood-derived mast cells only modestly enhanced MRGPRX2 surface levels and function (36). Thymic stromal lymphopoetin (TSLP) was recently shown to selectively enhance MRGPRX2-mediated degranulation of skin mast cells (37). This effect was mediated at the functional level which again emphasises the possibility of MRGPRX2 pathway enhancement beyond simple receptor expression level. Echoing the importance of the microenvironment to mast cell differentiation and functional responses, the culture of normally unresponsive mast cells in fibronectin or with fibroblasts has been shown to induce sensitivity to polybasic stimuli (38). Development of complex, yet more physiologically relevant mast cell culture systems (39), as well as proteomic (32) and transcriptomic (31, 33) characterisation from patient tissue samples will assist with better understanding the regulation of mast cell MRGPRX2 expression *in vivo*.

There is evidence in some disease states, including severe chronic urticaria (CSU) (35) and asthma (40), that MRGPRX2 levels on mast cells are elevated. A recent study has also provided functional evidence for enhanced MRGPRX2 activation in lesional biopsies taken from patients with ulcerative colitis compared to matched non-lesional controls (41). However, these conditions are not known to be strongly associated with increased susceptibility to drug-induced anaphylaxis.

A clearer understanding of patients with diagnosed mast cell disorders might also help clarify mechanisms leading to drug-induced, IgE-independent anaphylaxis. Whilst mastocytosis has been identified as a risk factor for a largely IgE-dependent anaphylaxis to *Hymenoptera* stings (42), evidence for enhanced drug-induced sensitivity is not as clear. A systematic review of reactions to invasive procedures in patients with mastocytosis, did indeed find an increased rate of reaction to drug exposure. Compared to the general population this varied from 5% in some studies, to 1% in larger studies (43), but significantly this rate was lower than anticipated for this population. However, in the surgical setting, patients with mast cells disorders are routinely given prophylactic drugs, including antihistamines and glucocorticoids, to protect from presumed reactions. This may then account for the relatively low incidence of drug-induced anaphylaxis recorded. Intriguingly, Deepak et al. have recently demonstrated enhanced MRGPRX2 expression in patients with maculopapular cutaneous mastocytosis (44). However, another study has shown that a lower burden of skin mast cells is a risk factor for anaphylaxis in systemic mastocytosis (45). This again reinforces the lack of clarity in the role that MRGPRX2 expression plays clinically in drug-induced anaphylaxis even in mast cell disease.

Mast cell models derived from patients who suffered acute drug-induced anaphylaxis would be a highly valuable tool to identify if elevated MRGPRX2 expression or function underpin the drug hypersensitivity. In such studies, CD34^+^ blood progenitor cells could be cultured into mature mast cells. Numerous methods exist, and these have been recently compared (36). A recent study has compared blood-derived mast cells from patients who had likely IgE-dependent with a possible MRGPRX2-dependent drug-induced anaphylaxis (46). Whilst the study was small in terms of patient numbers, interestingly, they could show no difference in reactivity to MRGPPRX2 agonists between the cohorts. Further work is needed to extend and confirm these findings. Whilst blood volumes might be a limitation to this targeted, patient-specific approach, single cell analytical methods, including analysis of mast cell function (47), increasingly make such limitations less challenging.

Recently, a new approach has been described where mast cells derived from human induced pluripotent stem cells (iPSCs) exhibit responsiveness to MRGPRX2 agonists (48). Previous studies using iPSC-derived mast cells have been reported, but these did not examine MRGPRX2 activation (49, 50). Whilst these studies used existing iPSC lines, they support the generation of patient-specific mast cells from those who experienced possible MRGPRX2-dependent, acute-drug-induced anaphylaxis. The extensive time and costs associated with this approach, again highlights the need to accurately characterize drug-responsive patients to ensure the utility of this endeavor.

### Polymorphisms in MRGPRX2 and/or Other Pathways That Might Heighten Mast Cell Responses to Drugs

Perhaps the most straightforward explanation behind the rare, proposed heightened sensitivity of some individuals to MRGPRX2-dependent anaphylaxis is receptor polymorphism. Given the incidence of drug-induced anaphylaxis, this polymorphism would have likely low penetrance. The GPCR database (GPCRdb.org) identifies numerous natural missense mutations in the MRGPRX2 protein coding region with predicted disruptive effects. Several studies have investigated these polymorphisms on MRGPRX2 activity. One study, examining some of the most common variants, revealed that all had neutral activity or demonstrated a loss of function to MRGPRX2 agonists (51). Importantly, the authors examined a range of agonists as studies have demonstrated evidence for biased agonism in MRGPRX2 activation (52, 53). Further studies have examined MRGPRX2 mutants where indeed some gain-of-function polymorphisms in the receptor C-terminal region were identified with modest enhancement of degranulation (54, 55). The clinical significance of these variants is however as yet unclear.

It is also possible that gene variants in mast cell signaling pathways underpin heightened sensitivity to MRGPRX2 agonists, increasing susceptibility to anaphylaxis. A precedent for this possibility is a rare PLCG2 variant that is associated with cold-induced mast cell activation and urticaria (56). Moreover, a recent study has identified diminished levels of PGE_2_ as a contributing factor to anaphylaxis (57). Whilst this study focused exclusively on clinical samples from likely IgE-dependent *Hymenoptera* sting-induced anaphylaxis, a deficiency in PGE2 levels would also be predicted to potentiate MRGPRX2 agonist-induced mast cell activation.

Clearly, more expansive genetic analyses are needed to correlate MRGPRX2 receptor or pathway polymorphism with clinical episodes of drug-induced acute anaphylaxis. Again, this connection will be greatly facilitated by improved clinical classification of presenting patients, and if shown, could be extraordinarily beneficial, given the potential rapid translation to predictive testing.

### Targeting MRGPRX2 for Therapeutic Benefit: Iterative Learning From Drug-Induced Anaphylaxis

**Figure 2:**
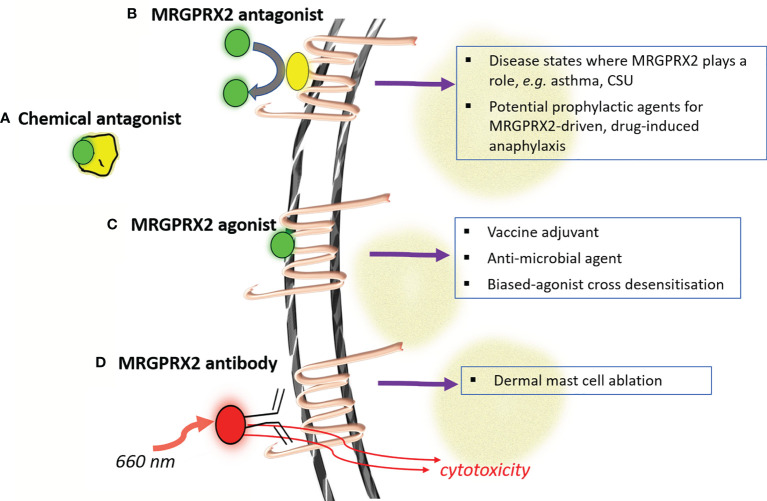
Modulating MRGPRX2 for putative therapeutic benefit. Four major strategies have been advanced for modulating the activity of mast cells through MRGPRX2 (labeled a-d). Antagonism at MRGPRX2 can be harnessed at both the ligand **(A)** and receptor **(B)** levels whilst complete or signaling-biased MRGPRX2 agonists **(C)** could be used in a number of settings to modulate immunity. Considerations around the safety of this later approach would be clarified through better understanding if/how MRGPRX2 contributes to acute drug-induced anaphylaxis. The relatively unique and high-expression levels of MRGPRX2 in skin mast cells has also been proposed as a strategy for antibody-targeted selective mast cell ablation **(D)**. (CSU- chronic spontaneous urticaria).

To this point, we have focused attention towards considering if and how MRGPRX2 contributes to drug-induced acute anaphylaxis. As proposed in **Figure 1**, further research is needed to establish this connection to an extent where it has clinically predictive value. This improved understanding will importantly also help inform the actual clinical risk of MRGPRX2 activation by novel drug candidates across the therapeutic spectrum and more clearly direct the proposed modulation of MRGPRX2 in a number of clinical settings. Whilst based on the discussion above, MRGPRX2 antagonists would seem of most clinical utility, the potential value of agonist drugs has also been examined. This makes clarification of the role of MRGPRX2 in drug-induced anaphylaxis of particular importance. Below, we summarise a number of current approaches to regulating MRGPRX2 activity (**Figure 2**), which has also been reviewed recently by others (5).

Chemical antagonism of NMBAs such as rocuronium by the reversal agent sugammadex has been proposed as a means of managing acute drug-induced anaphylaxis, although a consensus statement recommends against it (58). A recent study by our group has however shown inhibitory activity of sugammadex on some, but not all, endogenous activators of MRGPRX2 (59). Whilst speculative, this raises the possibility of using sugammadex, outside of the drugs conventional rocuronium-reversal role, to selectively modulate MRGPRX2 activation by endogenous agonists in certain disease states.

The selective expression of MRGPRX2 on mast cells has been recently harnessed for mast cell ablation. Utilising an anti-MRGPRX2 antibody conjugated to the compound IR700DX, which is activated by near infrared light exposure, Plum et al. demonstrated the depletion of dermal mast cells in a human skin explant model (32). This work exemplifies the innovative research that targets MRGPRX2 which could lead to new therapeutic approaches for mast cell-mediated disease.

A number of small molecule MRGPRX2 antagonists have been proposed/identified with many having relatively low potency and uncertain mechanism of antagonism (60–62). Recent compound screening efforts have identified some more potent and diverse agents however (63–65). As evidence continues to be established on the role of MRGPRX2 in inflammatory diseases of the skin and airways, it is likely that further momentum in this area will lead to compounds that could be envisaged to enter clinical development. Assuming an appropriate evidence-base, and predictive test, such compounds could in theory also serve as prophylactic agents to minimize MRGPRX2-dependent anaphylaxis risk, particularly in the perioperative setting.

MRGPRX2 agonists have shown potential as both vaccine adjuvants (66) and anti-microbial agents, both directly and through enhancing adaptive immunity (67, 68). Several new humanized mast cell mouse models have been developed that will better facilitate the predictive value of such studies to the human system (26, 69). Desensitisation of MRGPRX2 by agonists biased towards receptor internalization has also been proposed as a therapeutic option, particularly in cutaneous disorders where mast cells can be targeted topically (53). The broad safety of such an approach would benefit from a much clearer appreciation of MRGPRX2 gained through investigation of drug-induced anaphylaxis. It is plausible, for instance, that polymorphisms in MRGPRX2 and/or its downstream signaling might skew the nature of the biased agonism rendering this approach inappropriate at least in some.

### Summary and Conclusions

The number of clinically used drugs now known to act as MRGPRX2 agonists, at least in a laboratory setting, continues to expand. This reinforces the necessity of better understanding the role of MRGPRX2 in drug-induced anaphylaxis to determine if this receptor plausibly explains events where a clear connection to IgE sensitization cannot be made. In this review, we have discussed potential patient-specific factors that might account for rare and detrimental sensitivity. Throughout, the key value in developing better clinical stratification of patients experiencing drug-induced anaphylaxis, to highlight those likely to have a MRGPRX2 basis, has also been emphasised. This improved stratification, accompanied by more comprehensive cell, genomic and proteomic approaches are needed to firstly establish and secondly understand the basis of heightened patient MRGPRX2 responses. This knowledge could be key to predicting and hence avoiding these potentially devasting anaphylactic reactions. This insight will moreover better inform new drug development, establishing the real-world implications of MRGPRX2 agonism and moreover assisting in realising the full therapeutic potential of MRGPRX2 as a drug target.

## Author Contributions

All authors contributed to the writing, review, editing and final presentation of this manuscript.

## Funding

This work was in part supported by a research grant from the Australian and New Zealand College of Anaesthetists (ANZCA).

## Conflict of Interest

The authors declare that the research was conducted in the absence of any commercial or financial relationships that could be construed as a potential conflict of interest.

## Publisher’s Note

All claims expressed in this article are solely those of the authors and do not necessarily represent those of their affiliated organizations, or those of the publisher, the editors and the reviewers. Any product that may be evaluated in this article, or claim that may be made by its manufacturer, is not guaranteed or endorsed by the publisher.

## References

[1] Harper NJN Cook TM Garcez T Lucas DN Thomas M Kemp H. Anaesthesia, Surgery, and Life-Threatening Allergic Reactions: Management and Outcomes in the 6th National Audit Project (NAP6). Br J Anaesth (2018) 121:172–88. doi: 10.1016/j.bja.2018.04.015 29935569

[2] Reitter M Petitpain N Latarche C Cottin J Massy N Demoly P. Fatal Anaphylaxis With Neuromuscular Blocking Agents: A Risk Factor and Management Analysis. Allergy (2014) 69:954–9. doi: 10.1111/all.12426 24813248

[3] Ebo DG Clarke RC Mertes PM Platt PR Sabato V Sadleir PHM. Molecular Mechanisms and Pathophysiology of Perioperative Hypersensitivity and Anaphylaxis: A Narrative Review. Br J Anaesth (2019) 123:e38–49. doi: 10.1016/j.bja.2019.01.031 30916022

[4] Bruhns P Chollet-Martin S. Mechanisms of Human Drug-Induced Anaphylaxis. J Allergy Clin Immunol (2021) 147:1133–42. doi: 10.1016/j.jaci.2021.02.013 33832695

[5] Roy S Chompunud Na Ayudhya C Thapaliya M Deepak V Ali H. Multifaceted MRGPRX2: New Insight Into the Role of Mast Cells in Health and Disease. J Allergy Clin Immunol (2021) 148:293–308. doi: 10.1016/j.jaci.2021.03.049 33957166 PMC8355064

[6] Kühn H Kolkhir P Babina M Düll M Frischbutter S Fok JS. Mas-Related G Protein-Coupled Receptor X2 and Its Activators in Dermatologic Allergies. J Allergy Clin Immunol (2021) 147:456–69. doi: 10.1016/j.jaci.2020.08.027 33071069

[7] Thapaliya M Chompunud Na Ayudhya C Amponnawarat A Roy S Ali H. Mast Cell-Specific MRGPRX2: A Key Modulator of Neuro-Immune Interaction in Allergic Diseases. Curr Allergy Asthma Rep (2021) 21:3. doi: 10.1007/s11882-020-00979-5 33398613 PMC9595336

[8] McNeil BD Pundir P Meeker S Han L Undem BJ Kulka M. Identification of a Mast-Cell-Specific Receptor Crucial for Pseudo-Allergic Drug Reactions. Nature (2015) 519:237–41. doi: 10.1038/nature14022 PMC4359082 25517090

[9] Sinert R Levy P Bernstein JA Body R Sivilotti MLA Moellman J. Randomized Trial of Icatibant for Angiotensin-Converting Enzyme Inhibitor-Induced Upper Airway Angioedema. J Allergy Clin Immunol Pract (2017) 5:1402–9. doi: 10.1016/j.jaip.2017.03.003 28552382

[10] Baş M Greve J Stelter K Havel M Strassen U Rotter N. A Randomized Trial of Icatibant in ACE-Inhibitor-Induced Angioedema. N Engl J Med (2015) 372:418–25. doi: 10.1056/NEJMoa1312524 25629740

[11] Grimes J Desai S Charter NW Lodge J Moita Santos R Isidro-Llobet A. Mrgx2 is a Promiscuous Receptor for Basic Peptides Causing Mast Cell Pseudo-Allergic and Anaphylactoid Reactions. Pharmacol Res Perspect (2019) 7:e00547. doi: 10.1002/prp2.547 31832205 PMC6887720

[12] John LM Dalsgaard CM Jeppesen CB Conde-Frieboes KW Baumann K Knudsen NPH. *In Vitro* Prediction of *In Vivo* Pseudo-Allergenic Response *via* MRGPRX2. J Immunotoxicol (2021) 18:30–6. doi: 10.1080/1547691X.2021.1877375 33570451

[13] Finkelman FD Khodoun MV Strait R. Human IgE-Independent Systemic Anaphylaxis. J Allergy Clin Immunol (2016) 137:1674–80. doi: 10.1016/j.jaci.2016.02.015 PMC7607869 27130857

[14] Jönsson F de Chaisemartin L Granger V Gouel-Chéron A Gillis CM Zhu Q. An Igg-Induced Neutrophil Activation Pathway Contributes to Human Drug-Induced Anaphylaxis. Sci Transl Med (2019) 11:eaat1479s. doi: 10.1126/scitranslmed.aat1479 31292264

[15] Reber LL Hernandez JD Galli SJ. The Pathophysiology of Anaphylaxis. J Allergy Clin Immunol (2017) 140:335–48. doi: 10.1016/j.jaci.2017.06.003 PMC5657389 28780941

[16] Gowthaman U Chen JS Zhang B Flynn WF Lu Y Song W. Identification of a T Follicular Helper Cell Subset That Drives Anaphylactic IgE. Science (2019) 365:eaaw6433. doi: 10.1126/science.aaw6433 31371561 PMC6901029

[17] Drug Allergy: Diagnosis and Management. National Institute for Health and Care Excellence (NICE), Clinical Guideline [CG183]. Available at: https://www.nice.org.uk/guidance/cg183. 31841280

[18] Ebo DG van der Poorten ML Elst J Van Gasse AL Mertens C Bridts C. Immunoglobulin E Cross-Linking or MRGPRX2 Activation: Clinical Insights From Rocuronium Hypersensitivity. Br J Anaesth (2021) 126:e27–9. doi: 10.1016/j.bja.2020.10.006 33153719

[19] Noguchi S Takekawa D Saito J Hashiba E Hirota K. Serum Tryptase Cannot Differentiate Vancomycin-Induced Anaphylaxis From Red Man Syndrome. J Clin Immunol (2019) 39:855–6. doi: 10.1007/s10875-019-00707-3 31659619

[20] Varricchi G Pecoraro A Loffredo S Poto R Rivellese F Genovese A. Heterogeneity of Human Mast Cells With Respect to MRGPRX2 Receptor Expression and Function. Front Cell Neurosci (2019) 13:299. doi: 10.3389/fncel.2019.00299 31333418 PMC6616107

[21] Veien M Szlam F Holden JT Yamaguchi K Denson DD Levy JH. Mechanisms of Nonimmunological Histamine and Tryptase Release From Human Cutaneous Mast Cells. Anesthesiology (2000) 92:1074–81. doi: 10.1097/00000542-200004000-00026 10754628

[22] Ebo DG Bridts CH Mertens CH Sabato V. Principles, Potential, and Limitations of *Ex Vivo* Basophil Activation by Flow Cytometry in Allergology: A Narrative Review. J Allergy Clin Immunol (2021) 147:1143–53. doi: 10.1016/j.jaci.2020.10.027 33152367

[23] Wedi B Gehring M Kapp A. The Pseudoallergen Receptor MRGPRX2 on Peripheral Blood Basophils and Eosinophils: Expression and Function. Allergy (2020) 75:2229–42. doi: 10.1111/all.14213 32003863

[24] Sabato V Elst J Van Houdt M Bridts C Mertens C Ebo DG. Surface Expression of MRGPRX2 on Resting Basophils: An Area of Controversy. Allergy (2020) 75:2421–2. doi: 10.1111/all.14252 32929729

[25] Gaudenzio N Sibilano R Marichal T Starkl P Reber LL Cenac N. Different Activation Signals Induce Distinct Mast Cell Degranulation Strategies. J Clin Invest (2016) 126:3981–98. doi: 10.1172/JCI85538 PMC5096814 27643442

[26] Dispenza MC Krier-Burris RA Chhiba KD Undem BJ Robida PA Bochner BS. Bruton’s Tyrosine Kinase Inhibition Effectively Protects Against Human IgE-Mediated Anaphylaxis. J Clin Invest (2020) 130:4759–70. doi: 10.1172/JCI138448 PMC7456252 32484802

[27] Dispenza MC Pongracic JA Singh AM Bochner BS. Short-Term Ibrutinib Therapy Suppresses Skin Test Responses and Eliminates IgE-Mediated Basophil Activation in Adults With Peanut or Tree Nut Allergy. J Allergy Clin Immunol (2018) 141:1914–6. doi: 10.1016/j.jaci.2017.12.987 PMC7297295 29360526

[28] Bradding P Arthur G. Mast Cells in Asthma–State of the Art. Clin Exp Allergy (2016) 46:194–263. doi: 10.1111/cea.12675 26567481

[29] Metcalfe DD Baram D Mekori YA. Mast Cells. Physiol Rev (1997) 77:1033–79. doi: 10.1152/physrev.1997.77.4.1033 9354811

[30] Valent P Akin C Hartmann K Nilsson G Reiter A Hermine O. Mast Cells as a Unique Hematopoietic Lineage and Cell System: From Paul Ehrlich’s Visions to Precision Medicine Concepts. Theranostics (2020) 10:10743–68. doi: 10.7150/thno.46719 PMC7482799 32929378

[31] Dwyer DF Barrett NA Austen KF. Immunological Genome Project Consortium. Expression Profiling of Constitutive Mast Cells Reveals a Unique Identity Within the Immune System. Nat Immunol (2016) 17:878–87. doi: 10.1038/ni.3445 PMC5045264 27135604

[32] Plum T Wang X Rettel M Krijgsveld J Feyerabend TB Rodewald HR. Human Mast Cell Proteome Reveals Unique Lineage, Putative Functions, and Structural Basis for Cell Ablation. Immunity (2020) 52:404–16. doi: 10.1016/j.immuni.2020.01.012 32049054

[33] Jiang J Faiz A Berg M Carpaij OA Vermeulen CJ Brouwer S. Gene Signatures From Scrna-Seq Accurately Quantify Mast Cells in Biopsies in Asthma. Clin Exp Allergy (2020) 50:1428–31. doi: 10.1111/cea.13732 PMC7756890 32935368

[34] Patella V Marinò I Lampärter B Arbustini E Adt M Marone G. Human Heart Mast Cells. Isolation, Purification, Ultrastructure, and Immunologic Characterization. J Immunol (1995) 154:2855–65. 7533185

[35] Fujisawa D Kashiwakura J Kita H Kikukawa Y Fujitani Y Sasaki-Sakamoto T. Expression of Mas-Related Gene X2 on Mast Cells Is Upregulated in the Skin of Patients With Severe Chronic Urticaria. J Allergy Clin Immunol (2014) 134:622–33. doi: 10.1016/j.jaci.2014.05.004 24954276

[36] Elst J Sabato V van der Poorten MM Faber M Van Gasse AL De Puysseleyr LP. Peripheral Blood Cultured Mast Cells: Phenotypic and Functional Outcomes of Different Culture Protocols. J Immunol Methods (2021) 492:113003. doi: 10.1016/j.jim.2021.113003 33647250

[37] Babina M Wang Z Franke K Zuberbier T. Thymic Stromal Lymphopoietin Promotes MRGPRX2-Triggered Degranulation of Skin Mast Cells in a STAT5-Dependent Manner With Further Support From JNK. Cells (2021) 10:102. doi: 10.3390/cells10010102 PMC7826995 33429916

[38] Swieter M Hamawy MM Siraganian RP Mergenhagen SE. Mast Cells and Their Microenvironment: The Influence of Fibronectin and Fibroblasts on the Functional Repertoire of Rat Basophilic Leukemia Cells. J Periodontol (1993) 64:492–6. 7686221

[39] Ozpinar EW Frey AL Arthur GK Mora-Navarro C Biehl A Snider DB. Dermal Extracellular Matrix-Derived Hydrogels as an *In Vitro* Substrate to Study Mast Cell Maturation. Tissue Eng Part A (2020) 27:1008–22. doi: 10.1089/ten.TEA.2020.0142 PMC8403210 33003982

[40] Manorak W Idahosa C Gupta K Roy S Panettieri R Jr Ali H. Upregulation of Mas-Related G Protein Coupled Receptor X2 in Asthmatic Lung Mast Cells and its Activation by the Novel Neuropeptide Hemokinin-1. Respir Res (2018) 19(1):1. doi: 10.1186/s12931-017-0698-3 29295703 PMC5751818

[41] Chen E Chuang LS Giri M Villaverde N Hsu NY Sabic K. Inflamed Ulcerative Colitis Regions Associated With MRGPRX2-Mediated Mast Cell Degranulation and Cell Activation Modules, Defining a New Therapeutic Target. Gastroenterology (2021) 160:1709–24. doi: 10.1053/j.gastro.2020.12.076 PMC8494017 33421512

[42] Gülen T Oude Elberink JNG Brockow K. Anaphylaxis in Mastocytosis (Chapter 9). In: Akin C, editor. Mastocytosis: A Comprehensive Guide. Cham: Springer (2020).

[43] Hermans MAW Arends NJT Gerth van Wijk R van Hagen PM Kluin-Nelemans HC Oude Elberink HNG. Management Around Invasive Procedures in Mastocytosis. Ann Allergy Asthma Immunol (2017) 199:304–9. doi: 10.1016/j.anai.2017.07.022 28866309

[44] Deepak V Komarow HD Alblaihess AA Carter MC Metcalfe DD Ali H. Expression of MRGPRX2 in Skin Mast Cells of Patients With Maculopapular Cutaneous Mastocytosis. J Allergy Clin Immunol Pract (2021) 9:3841–3.e1. doi: 10.1016/j.jaip.2021.05.042 PMC8511159 34182161

[45] Gülen T Ljung C Nilsson G Akin C. Risk Factor Analysis of Anaphylactic Reactions in Patients With Systemic Mastocytosis. J Allergy Clin Immunol Pract (2017) 5:1248–55. doi: 10.1016/j.jaip.2017.02.008 28351784

[46] Elst J Maurer M Sabato V Faber MA Bridts CH Mertens C. Novel Insights on MRGPRX2-Mediated Hypersensitivity to Neuromuscular Blocking Agents and Fluoroquinolones. Front Immunol (2021) 12:668962. doi: 10.3389/fimmu.2021.668962 34385999 PMC8353374

[47] Folkerts J Gaudenzio N Maurer M Hendriks RW Stadhouders R Tam SY. Rapid Identification of Human Mast Cell Degranulation Regulators Using Functional Genomics Coupled to High-Resolution Confocal Microscopy. Nat Protoc (2020) 15:1285–310. doi: 10.1038/s41596-019-0288-6 PMC7197894 32060492

[48] Luo Y Vallone VF He J Frischbutter S Kolkhir P Moñino-Romero S. A Novel Approach for Studying Mast Cell-Driven Disorders: Mast Cells Derived From Induced Pluripotent Stem Cells. J Allergy Clin Immunol (2021) 6:S0091–6749(21)01201-X. doi: 10.1016/j.jaci.2021.07.027 34371081

[49] Igarashi A Ebihara Y Kumagai T Hirai H Nagata K Tsuji K. Mast Cells Derived From Human Induced Pluripotent Stem Cells are Useful for Allergen Tests. Allergol Int (2018) 67(2):234–42. doi: 10.1016/j.alit.2017.08.008 28919488

[50] Ikuno T Ito S Inoue T. Human Induced Pluripotent Stem Cell-Derived Mast Cells Useful for *In Vitro* Mast Cell Activation Assay Exhibiting Phenotypes and Morphological Characteristics of Human Mast Cells. J Toxicol Sci (2019) 44(11):789–97. doi: 10.2131/jts.44.789 31708535

[51] Alkanfari I Gupta K Jahan T Ali H. Naturally Occurring Missense MRGPRX2 Variants Display Loss of Function Phenotype for Mast Cell Degranulation in Response to Substance P, Hemokinin-1, Human Beta-Defensin-3, and Icatibant. J Immunol (2018) 201:343–9. doi: 10.4049/jimmunol.1701793 PMC6039248 29794017

[52] Roy S Ganguly A Haque M Ali H. Angiogenic Host Defense Peptide AG-30/5C and Bradykinin B_2_ Receptor Antagonist Icatibant Are G Protein Biased Agonists for MRGPRX2 in Mast Cells. J Immunol (2019) 202:1229–38. doi: 10.4049/jimmunol.1801227 PMC6369923 30651343

[53] Babina M Wang Z Roy S Guhl S Franke K Artuc M. MRGPRX2 is the Codeine Receptor of Human Skin Mast Cells: Desensitization Through Beta-Arrestin and Lack of Correlation With the FcεRI Pathway. J Invest Dermatol (2020) 141:1286–96.e4. doi: 10.1016/j.jid.2020.09.017 PMC8041898 33058860

[54] Chompunud Na Ayudhya C Roy S Alkanfari I Ganguly A Ali H. Identification of Gain and Loss of Function Missense Variants in MRGPRX2’s Transmembrane and Intracellular Domains for Mast Cell Activation by Substance P. Int J Mol Sci (2019) 20(21). doi: 10.3390/ijms20215247 PMC6862462 31652731

[55] Chompunud Na Ayudhya C Amponnawarat A Roy S Oskeritzian CA Ali H. MRGPRX2 Activation by Rocuronium: Insights From Studies With Human Skin Mast Cells and Missense Variants. Cells (2021) 10:156. doi: 10.3390/cells10010156 33467419 PMC7830812

[56] Ombrello MJ Remmers EF Sun G Freeman AF Datta S Torabi-Parizi P. Cold Urticaria, Immunodeficiency, and Autoimmunity Related to PLCG2 Deletions. N Engl J Med (2012) 366:330–8. doi: 10.1056/NEJMoa1102140 PMC3298368 22236196

[57] Rastogi S Willmes DM Nassiri M Babina M Worm M. PGE_2_ Deficiency Predisposes to Anaphylaxis by Causing Mast Cell Hyperresponsiveness. J Allergy Clin Immunol (2020) 146:1387–96. doi: 10.1016/j.jaci.2020.03.046 32407837

[58] Garvey LH Dewachter P Hepner DL Mertes PM Voltolini S Clarke R. Management of Suspected Immediate Perioperative Allergic Reactions: An International Overview and Consensus Recommendations. Br J Anaesth (2019) 123:e50–64. doi: 10.1016/j.bja.2019.04.044 31130272

[59] Fernandopulle NA Zhang SS Soeding PF Mackay GA. MRGPRX2 Activation in Mast Cells by Neuromuscular Blocking Agents and Other Agonists: Modulation by Sugammadex. Clin Exp Allergy (2020) 51:685–95. doi: 10.1111/cea.13801 33275825

[60] Ding Y Che D Li C Cao J Wang J Ma P. Quercetin Inhibits Mrgprx2-Induced Pseudo-Allergic Reaction *via* Plcγ-IP3R Related Ca^2+^ Fluctuations. Int Immunopharmacol (2019) 66:185–97. doi: 10.1016/j.intimp.2018.11.025 30471617

[61] Callahan BN Kammala AK Syed M Yang C Occhiuto CJ Nellutla R. Osthole, a Natural Plant Derivative Inhibits MRGPRX2 Induced Mast Cell Responses. Front Immunol (2020) 11:703. doi: 10.3389/fimmu.2020.00703 32391014 PMC7194083

[62] Kumar M Singh K Duraisamy K Allam A A Ajarem J Kwok Chong Chow B. Protective Effect of Genistein Against Compound 48/80 Induced Anaphylactoid Shock *via* Inhibiting MAS Related G Protein-Coupled Receptor X2 (MRGPRX2). Molecules (2020) 25:1028. doi: 10.3390/molecules25051028 PMC7179155 32106575

[63] Suzuki Y Liu S Ogasawara T Sawasaki T Takasaki Y Yorozuya T. A Novel MRGPRX2-Targeting Antagonistic DNA Aptamer Inhibits Histamine Release and Prevents Mast Cell-Mediated Anaphylaxis. Eur J Pharmacol (2020) 878:173104. doi: 10.1016/j.ejphar.2020.173104 32320700

[64] Ogasawara H Furuno M Edamura K Noguchi M. Novel MRGPRX2 Antagonists Inhibit Ige-Independent Activation of Human Umbilical Cord Blood-Derived Mast Cells. J Leukoc Biol (2019) 106:1069–77. doi: 10.1002/JLB.2AB1018-405R 31299111

[65] Dondalska A Rönnberg E Ma H Pålsson SA Magnusdottir E Gao T. Amelioration of Compound 48/80-Mediated Itch and LL-37-Induced Inflammation by a Single-Stranded Oligonucleotide. Front Immunol (2020) 11:559589. doi: 10.3389/fimmu.2020.559589 33101278 PMC7554336

[66] Johnson-Weaver B Choi HW Abraham SN Staats HF. Mast Cell Activators as Novel Immune Regulators. Curr Opin Pharmacol (2018) 41:89–95. doi: 10.1016/j.coph.2018.05.004 29843056 PMC6448149

[67] Pundir P Liu R Vasavda C Serhan N Limjunyawong N Yee R. A Connective Tissue Mast-Cell-Specific Receptor Detects Bacterial Quorum-Sensing Molecules and Mediates Antibacterial Immunity. Cell Host Microbe (2019) 26:114–22. doi: 10.1016/j.chom.2019.06.003 PMC6649664 31278040

[68] Arifuzzaman M Mobley YR Choi HW Bist P Salinas CA Brown ZD. MRGPR-Mediated Activation of Local Mast Cells Clears Cutaneous Bacterial Infection and Protects Against Reinfection. Sci Adv (2019) 5:eaav0216. doi: 10.1126/sciadv.aav0216 30613778 PMC6314830

[69] Mencarelli A Gunawan M Yong KSM Bist P Tan WWS Tan SY. A Humanized Mouse Model to Study Mast Cells Mediated Cutaneous Adverse Drug Reactions. J Leukoc Biol (2020) 107:797–807. doi: 10.1002/JLB.3MA1219-210RR 31922289 PMC7322799

